# Comparison of the on-line effects of different motor simulation conditions on corticospinal excitability in healthy participants

**DOI:** 10.1038/s41598-021-92591-4

**Published:** 2021-06-23

**Authors:** C. Pfenninger, S. Grosprêtre, A. Remontet, T. Lapole

**Affiliations:** 1 Univ Lyon, UJM-Saint-Etienne, Inter-university Laboratory of Human Movement Biology, EA 7424, 40023 Saint-Etienne, France; 2 Univ Bourgogne Franche Comté, Culture, Sport, Health, Society, EA 4660 C3S, Besançon, France

**Keywords:** Neurophysiology, Neuroscience

## Abstract

In healthy participants, corticospinal excitability is known to increase during motor simulations such as motor imagery (MI), action observation (AO) and mirror therapy (MT), suggesting their interest to promote plasticity in neurorehabilitation. Further comparing these methods and investigating their combination may potentially provide clues to optimize their use in patients. To this end, we compared in 18 healthy participants abductor pollicis brevis (APB) corticospinal excitability during MI, AO or MT, as well as MI combined with either AO or MT. In each condition, 15 motor-evoked potentials (MEPs) and three maximal M-wave were elicited in the right APB. Compared to the control condition, mean normalized MEP amplitude (i.e. MEP/M) increased during MI (P = .003), MT (P < .001) and MT + MI (P < .001), without any difference between the three conditions. No MEP modulation was evidenced during AO or AO + MI. Because MI provided no additional influence when combined with AO or MT, our results may suggest that, in healthy subjects, visual feedback and unilateral movement with a mirror may provide the greatest effects among all the tested motor simulations.

## Introduction

Neuroplasticity can be facilitated by increasing primary motor cortex (M1) excitability^1^, which can be observed when performing voluntary movements. Yet, stroke patients, for example, may not be able to voluntarily activate their impaired muscles. In such cases, neurorehabilitation may rely on the use of motor simulation to activate M1 without performing actions^1,2^. While mirror therapy (MT) has demonstrated many benefits in this context^3^, motor imagery (MI) and action observation (AO) have recently emerged as potentially effective interventions in neurorehabilitation^4^ and have accordingly shown interesting perspectives for improving motor function in stroke patients^5,6^.

MI is a motor simulation method where participants imagine themselves performing the action, with an internal generation of visual and kinesthetic aspects of the movement but without any movement generation^7^. Motor imagery and physical practice share similar cortical areas activation, in particular motor areas^1^. As a consequence, studies using transcranial magnetic stimulation (TMS) in healthy participants demonstrated an increase in motor-evoked potential (MEP) amplitude during MI in the first dorsal interosseous (FDI)^8,9^, the abductor pollicis brevis (APB)^9,10^, and the flexor carpi radialis (FCR)^11,12^ muscles. This suggests an increased corticospinal excitability during imagery. It is then thought that MI may represent an intermediate level of cortical activity between rest and actual contraction^13^.

AO is another process of motor simulation where participants observe a movement performed by another person, without performing it. As with MI, AO activates similar regions of the brain as activated by physical practice^14^. Observation of upper limb movement has been reported to increase corticospinal excitability in healthy participants for the biceps^15^, the FDI^16–18^ and the flexor digitorum superficialis^19^ muscles. It is possible to further combine the two practices of AO and MI with « imagining the physiological sensations and kinesthetic experiences of action, and synchronizing this motor simulation with the congruent observed action »^7^. The visual stimulus provided by AO allows participants to focus on the movement’s sensations without the constraint of generating a visual mental image^20,21^. As a result, AO combined with MI has been reported to increase corticospinal excitability significantly more compared to AO or MI performed independently^22–25^.

In neurorehabilitation, mirror therapy (MT), which has been first described to treat phantom limb pain by Ramachandran in 1994^26^ is commonly used rather than MI or AO. During MT, patients move their unaffected limb in front of a mirror placed midsagittaly between the two limbs and observe the movement reflected in the mirror. MT can increase corticospinal excitability in the hemisphere ipsilateral to the limb in movement, as has been shown in healthy participants by greater MEP amplitude during MT compared to a control condition in FCR^27,28^, FDI^23,29,30^ and APB^31^ muscles. MT is thought to increase corticospinal excitability due to the combination of AO of participants’ own limb and voluntary unimanual movements^29,32,33^. Interestingly, a greater increase in corticospinal excitability is found when MT was combined with motor imagery, i.e. when participants were watching the movements of the hand in the mirror in the same time they had to imagine practicing the movement with the hidden hand^25,28^.

To optimize neurorehabilitation, it is crucial to compare how different motor simulation strategies may influence corticospinal excitability and how their combination may lead to further increases in excitability. With this objective in mind, preliminary data on healthy participants are necessary to decipher the effect of different combinations of MI, AO and MT before implementation of such interventions in patients. Therefore, we compared, in healthy participants APB MEP modulation during MI, AO or MT, as well as the combination of MI with either AO or MT. It was hypothesized that MI, AO and MT would improve corticospinal excitability in a similar manner. A second hypothesis was that combining MI with either AO or MT would facilitate corticospinal excitability more than AO or MT performed alone. This is, to date, the first work to establish in a single study design a general picture of the effects of all these different therapeutic approaches targeting neural plasticity, while most of the previous literature focused on one or two modalities.

## Materials and methods

### Participants

Eighteen healthy volunteers (15 men and 3 women; age: 27.9 ± 6.8 year; stature: 174.5 ± 9.2 cm; mass: 69.3 ± 12.7 kg) participated in the study. All participants were free from neurological illness or musculoskeletal injury, and had no contraindications to TMS^34^. Each participant completed the revised version of the Movement Imagery Questionnaire^35^ to determine self-estimation of MI ability. The mean MIQ-R score was 39.2 ± 6.8 (maximum score: 56), indicating a good imagery capacity. The study was approved by the University of Saint-Etienne ethics committee and conformed to the *Declaration of Helsinki*. Written informed consent was obtained from each participant prior to the study taking place.

### Experimental design

**Figure 1:**
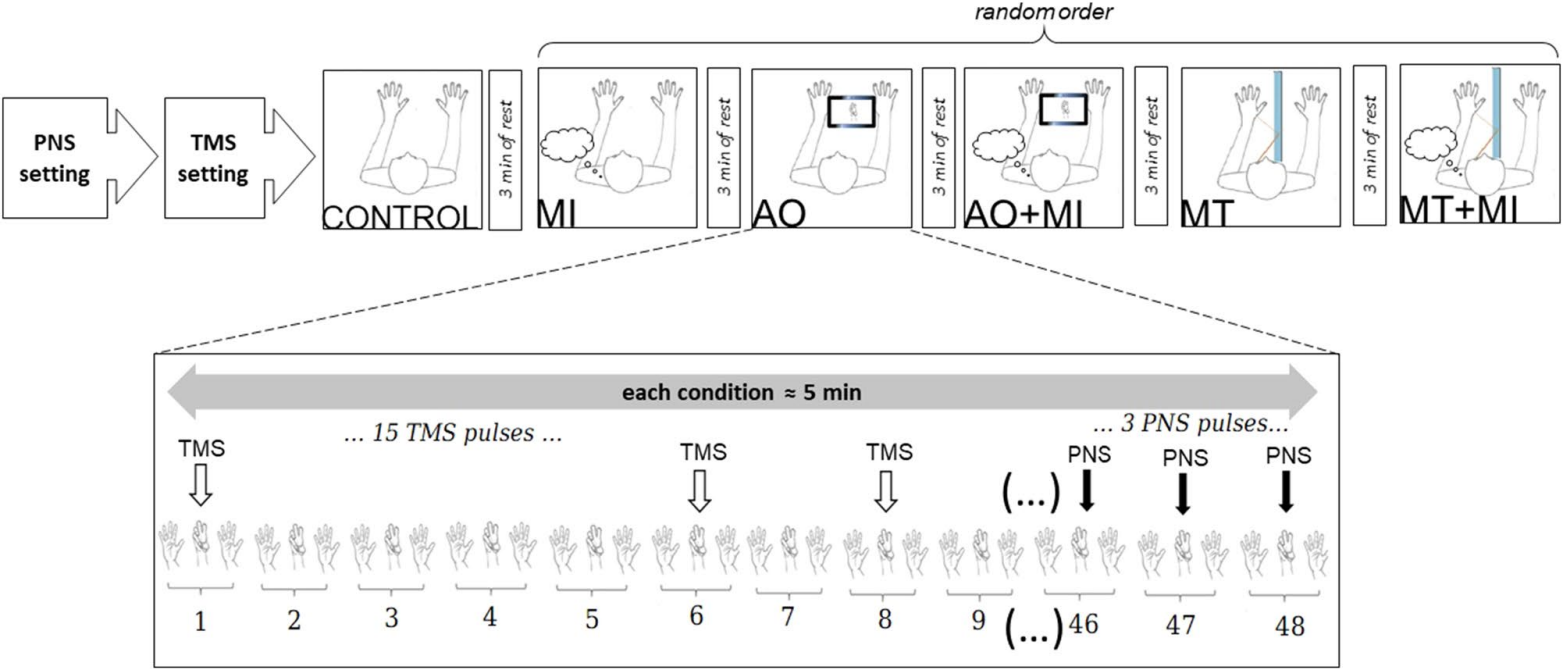
Overview of the experimental protocol. Series of fifteen magnetic (white arrows) and three peripheral nerves stimulations (black arrows) were performed for each condition about approximately five minutes (MI, AO, AO + MI, MT, MT + MI) separated by three minutes of rest. Control condition was performed at the beginning of the experiment.

Participants visited the laboratory once for an experimental session lasting around 90 min. They were placed comfortably in a secluded room to facilitate concentration for the entire duration of the experiment. After familiarization with the selected movement, optimal position and intensities of stimulation were determined for peripheral nerve stimulation (PNS) and TMS techniques (see below) to evoke responses on the right APB muscle. Then, a first series of 15 TMS pulses was delivered in a relaxed state to assess baseline corticospinal excitability, i.e. control condition without any MI, followed by three PNS stimulation to record maximal M-wave responses. Thereafter, a series of 15 TMS pulses followed by three PNS stimulations were delivered during the following randomized conditions: MI, AO, MT, AO + MI, MT + MI (Fig. 1—see below for detailed description). Note that we chose to perform the control condition before all other conditions to avoid potential carry-over effects between conditions^22^. In total, six conditions were recorded and analyzed. Each condition was separated by three minutes of rest. All the measurements were performed on the right hand.

### Protocol

The selected movement was an abduction of the thumb and fifth finger of the right hand, followed by adduction. The whole movement lasted five sec, with two sec of abduction, one sec of pinch and two sec of adduction. During AO, participants had to watch a video of an individual performing this movement on their right hand. The video was displayed on a laptop placed in front of participants. During the video, participants were instructed to watch it as it was a film, and importantly not to imagine performing the movement nor to perform it. During MT, a mirror was placed sagittally between participants’ arms. Participants were instructed to perform the movement with their left hand and focus on the reflected movement on the mirror giving the illusion of a movement on their right hand without imagining performing it. During MI, participants were instructed not to execute the movement but to imagine and feel the contractions and sensations that it would have produced (i.e. kinesthetic MI) while following series of metronome sounds. Auditive clues were provided every second with a higher tone to indicate the pinch phase. When MI was combined with AO participants had to imagine the movement with the same timing as indicated by the video.

For each of the five aforementioned conditions, a total of 45 repetitions was performed, with no rest in-between (Fig. 1). One TMS pulse was randomly delivered within each subset of three repetitions to avoid participants anticipation^22^. The TMS pulse was delivered during the pinch phase, i.e. slightly after two sec of movement onset. For the MI condition the participant learned to perform the movement and synchronize it to the auditive clues provided by the metronome. If the participant was out of sync with the metronome sounds or if the stimulation was not sent during the pinch phase, time was taken to regain the correct rhythm. For MT and AO conditions, TMS pulses were manually sent when the pinch phase was observed by the experimenter. At the end of each condition, three additional PNS stimulations were delivered during the pinch phase (i.e. as for TMS measurements) during three additional movement, with six sec of rest in-between. For the control condition, performed at the beginning of the protocol, 15 TMS pulses were delivered at seven sec of interval and followed by three PNS stimulations.

### EMG

Participants were first prepared by shaving, gently abrading the skin, and then cleaning it with isopropyl alcohol. EMG of the abductor pollicis brevis (APB) was recorded with a pair of self-adhesive surface electrodes (Meditrace 100, Covidien, Mansfield, MA) in a belly-tendon montage. Signal was analogue-to-digitally converted at a sampling rate of 2000 Hz by Powerlab system (16/30-ML880/P, ADInstruments, Bella Vista, Australia) and bio-amplifier (ML138, ADInstruments; common mode rejection ration = 85 db, gain = 5000) with bandpass filter (10–500 Hz) and analyzed offline using Labchart 7 software (ADInstruments)^36^.

### TMS

Single-pulse TMS was delivered to the left motor cortex using a figure-of-eight coil connected to a Magstim 200 magnetic stimulator (Magstim Co., Ltd., Whitland, UK). The coil was positioned over the right-hand area of the motor cortex. The coil was held tangentially to the scalp, with the handle pointing backwards and sideways (at a 45° angle from the midline) to induce a posterio-anterior current. Optimal coil position was identified as the position evoking the greatest APB MEP amplitude in response to a TMS pulse at 50% of maximal stimulator output. Once identified, this position was marked directly on a swimming pool cap worn by participants to ensure consistent placement throughout the experiment.

While TMS pulse intensity is classically defined as a percentage of the resting motor threshold^17–19^, it has been recently demonstrated that an intensity eliciting MEPs around 70% of maximal MEP amplitude is optimal to demonstrate modulation in corticospinal excitability during motor imagery^13^. To this end, we first performed an input–output curve where APB MEPs were recorded. Three consecutive TMS pulses at 10-s intervals were sent at each of the following randomly ordered stimulus intensities: 50%, 60%, 70%, 80% of maximal stimulator output. If APB MEP amplitudes did not reach a plateau with these intensities (i.e. if MEP amplitude was still increasing between 70 and 80% of maximal stimulator output), higher TMS intensities (i.e. 90% and 100% of maximal stimulator output) were used. After calculating the maximal APB MEP amplitude from this input–output curve, optimal intensity was determined from this curve as the one corresponding to approximately 70% of this maximal amplitude^13^. Three stimulations at this intensity were delivered to ensure that we were effectively close to 70% of maximal MEP amplitude, and intensity was adjusted if necessary. Then this intensity was kept constant throughout the whole experimental session.

### Peripheral nerve stimulation

An electrical stimulator (DS7A, Digitimer, Welwyn Garden City, Hertfordshire, UK) was used to stimulate the right median nerve. Single electrical stimuli of 0.2 ms duration were delivered through a bipolar bar stimulating electrode with 30 mm anode–cathode spacing (Bipolar Felt Pad Stimulating Electrode Part Number E.SB020/4 mm, Digitimer) placed on the median nerve at the wrist. Single stimuli were delivered incrementally (i.e. by 5-mA steps) until eliciting maximal M-wave on the APB muscle. The optimal intensity was then increased by 40% to confirm supramaximal stimulation.

### Analysis

The peak-to-peak amplitudes of the MEPs recorded in each condition were measured and normalized by the maximal M-wave amplitude recorded at the end of each condition. Trials with visual EMG activity prior to TMS stimulation were discarded. In addition, to monitor background EMG activity before TMS pulses, EMG traces were rectified and the mean amplitude between 75 and 50 ms before the onset of TMS was calculated and normalized to the maximal M-wave amplitude. When background EMG amplitude was superior to mean ± 1 standard deviation of the EMG amplitude calculated in the control condition, trials were discarded to ensure no effects of EMG activity on MEP amplitude^37^. However, the number of trials with such background activity has been accounted and compared between conditions.

### Statistical analysis

Statistical analysis was performed with Statistica software (StatSoft Inc., Tulsa, OK). All descriptive statistics presented in the text are mean values (± SD).

Because data normality was not respected (Shapiro–Wilk normality test) for the dataset related to the number of analysed MEPs, a Friedman non-parametric test was performed to compare all conditions. A post-hoc Wilcoxon test with Bonferroni correction for multiple comparisons (i.e. p values were multiplied by 15 since we had 15 comparisons) was then used when the Friedman test identified significant differences. For MEP amplitudes, data were logarithmically transformed for further analysis. Data normality (P = 0.19) and equality of variance (P = 0.86) were verified. Mauchly’s test [χ^2^ (14) = 19.28, P = 0.12] did not indicate any violation of sphericity. A one-way repeated measure ANOVA was performed to compare the mean normalized MEP amplitude between conditions. A post-hoc Bonferroni test for multiple comparisons was then used when the one-way repeated measure ANOVA identified significant differences. Statistical significance was set at P < 0.05.

For the comparison between conditions, we reported Cohen’s dav (d) effect size with d = 0.2, d = 0.5 and d = 0.8 used as small, medium and large effects, respectively^38^.

### Ethics approval

All procedures performed in studies involving human participants were in accordance with the University of Saint-Etienne ethics committee and with the 1964 Helsinki declaration and its later amendments or comparable ethical standards.

### Consent to participate

Informed consent was obtained from all individual participants included in the study.

### Consent for publication

Informed consent was obtained from all individual participants included in the study.

## RESULTS

After EMG processing, the mean normalized EMG was 0.002% (± 0.002). From the 15 MEPs recorded on each condition, we performed statistical analyses on 11.7 ± 1.7 MEPs for control condition, 9.1 ± 3.8 for MI, 11.1 ± 4 for AO, 10.8 ± 3.6 for AO + MI, 8.1 ± 3.9 for MT and 6.8 ± 4.4 for MT + MI. The Friedman test showed a significant difference between conditions (χ^2^ (5) = 23.7, P = 0.0003). Post hoc analysis demonstrated a significantly lower number of MEP during MT + MI (Z = 3.39, P = 0.009, d = 1.45) than for the control condition.

**Figure 2:**
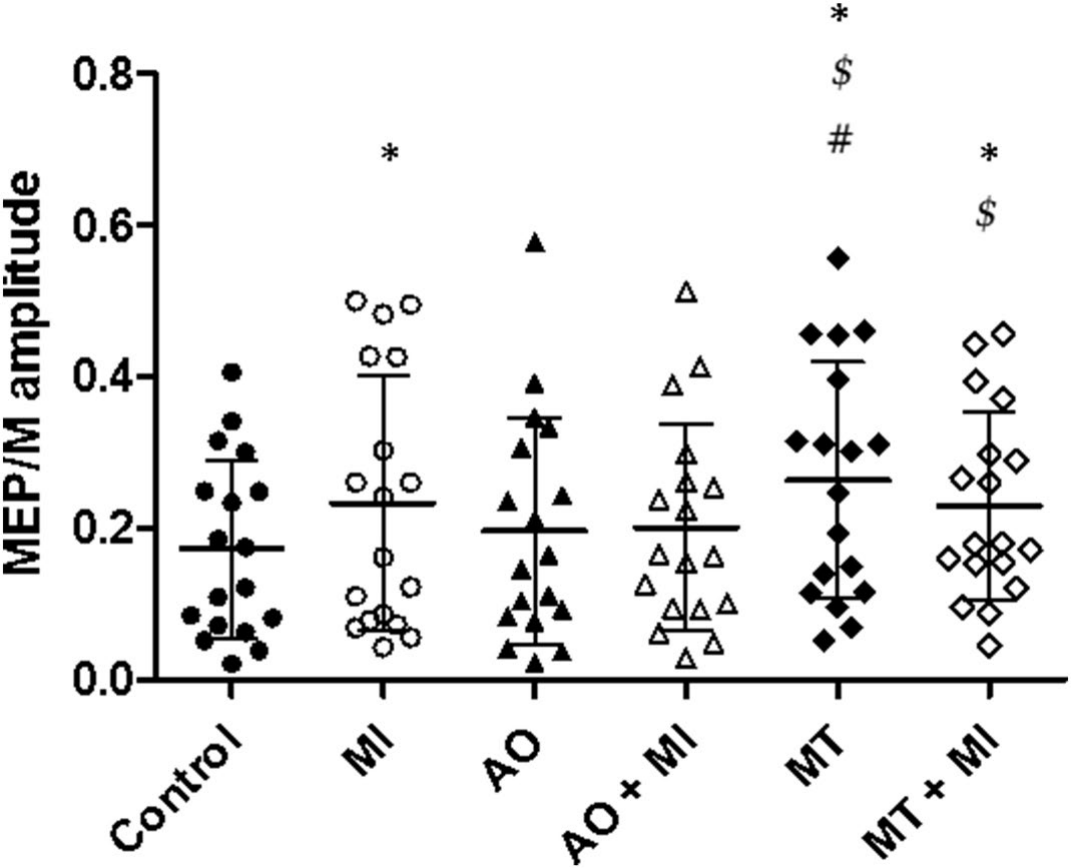
Comparison of normalized MEP amplitude between conditions. Individual data as well as group mean and standard deviation are presented for control, motor imagery (MI), action observation (AO), AO + MI, mirror therapy (MT) and MT + MI conditions. * indicates significant difference. $ indicates significant difference compared to AO. # indicates significant difference compared to AO + MI.

Mean normalized MEP amplitudes across the six experimental conditions are illustrated on Fig. 2. The one-way repeated measure ANOVA showed a significant difference between conditions (F_(6,12)_ = 18.7, P < 0.0001, η^2^_p_ = 0.9). Bonferroni post-hoc tests demonstrated a marked increase in MEP amplitude during MI (t = 3.88, P = .003, d = 0,42), MT (t = 6.75, P < 0.001, d = 0.66) and MT + MI (t = 5.52, P < 0.001, d = 0.47) compared to the control condition. There were no significant differences between AO (t = 1.28, P = 0.1, d = 0.18), AO + MI (t = 2.54, P = 0.19, d = 0.23) and control condition. MEP amplitude during MT was greater than for AO (t = 5.48, P < 0.001, d = 0.44) and AO + MI (t = 4.2, P < 0.001, d = 0.42) but was not different when compared to MI (t = 2.87, P = 0.08, d = 0.66) or MT + MI (t = 1.23, P = 1, d = 0.24). There was also a significant difference between MT + MI and AO (t = 4.24, P < 0.001, d = 0.47).

## Discussion

The objective of our study was to compare for the APB muscle the corticospinal excitability modulation provided by MI, AO and MT, and whether the combination of the two latter with MI would provide greater modulation than AO or MT alone. Our results demonstrated that MEP modulation was similarly observed during MI, MT and MT + MI. In contrast, no differences with the control condition were observed during the other motor simulation conditions.

### Facilitation of corticospinal excitability during motor imagery performed alone

The effects of MI on corticospinal excitability have been investigating for many years showing increases in MEP amplitude when compared to a control condition^9,11,39^. In our study, the mean normalized MEP amplitude was 40% higher during MI without any visual feedback than during the control condition, agreeing with previous findings. For instance, for the same muscle as we investigated, Kiers and al. (1997) study reported a mean 25% increase in MEP amplitude during MI of thumb abduction^11^. Yet, it should be acknowledged that Kang and al. (2011) conversely did not find any increase in corticospinal excitability during MI of the same movement^25^. The origin of discrepancies between studies could be due to experimental differences such as the intensity of TMS, timing of stimulation or timing of the imagined movement. Differences in how participants performed motor imagery can also explain differences between studies. For instance, no specific instructions were provided to the participants in the aforementioned studies while it is known that kinesthetic imagery is more effective than visual imagery^39^.

Increased MEP amplitude during MI is thought to result from the activation of motor areas that are involved in the imagined movement^1^. As such, MI may represent an intermediate level of cortical activity between rest and actual contraction. The corresponding stimulation of the corticospinal pathways during MI is then either under the threshold of spinal motoneurons excitability or inhibited in the lower stages of the nervous system, thus preventing from muscular contraction^40,41^. Another hypothesis to explain the increase in MEP amplitude during MI would be the influence that attentional processing may have on MEP modulation. For instance, corticospinal excitability can be increased “simply” because of the presence of cognitive activity during MI and not necessarily because of MI per se^24^. It has accordingly been demonstrated that MI did not induce any MEP modulation when compared to purely cognitive tasks such as backward counting^24,42,43^. In the context of the present study, no such condition was included and it could be questioned to what extent the use of a metronome during our MI condition may have influenced the results by further increasing attentional processing of our participants, which may be supported by the lack of effects when MI was combined to AO or MT (see below).

### Corticospinal excitability during action observation performed alone or in combination with motor imagery

While a majority of studies found an increase in MEP amplitude during AO^15–17^, we did not find any change in MEP amplitude compared to the control condition in the present study. However, this is not an isolated finding^29,44–46^. For instance, Brighina and al. (2000) did not find any increase in MEP amplitude in the APB muscle during observation of a single movement (i.e. thumb abduction as in the present study) while an increase was reported during a more complex motor sequence performed with different fingers^47^. The effects of AO are thought to be mediated by the mirror neuron system. This group of neurons is similarly activated during the execution or the observation of the same action^48^. The activation of the mirror neuron system increases motor cortex excitability^48^. Interestingly, this system seems to be activated to a greater extent during observation of a goal-directed movement than during a movement out of context^16,17,49^. It is thus plausible that the chosen movement was not complex enough and too much decontextualized to sufficiently activate the mirror neuron system and consequently increase corticospinal excitability.

When combining AO with MI, we surprisingly did not observe any further increases in corticospinal excitability when compared to AO performed alone or to the control condition. This finding is not consistent with previous research into the effects of combined AO and MI on corticospinal excitability^7,44–46,50^. For example, in the FDI muscle, the majority of studies found a greater increase in MEP amplitude during AO combined with MI than AO or MI alone^22,24,51^. Yet, in opposition to the latter studies, AO did not increase corticospinal excitability in our study and it remains difficult to elucidate whether the MEP modulation during MI was a true effect of MI or whether it was related to the cognitive load provided by the use of a metronome. Although speculative, the latter hypothesis could be supported by the fact that when MI was combined with AO and the metronome was no more used (i.e. subjects had to mentally imagine the movement while following the rhythm imposed by the video), no MEP modulation was observed. Interestingly, a recent study suggested that MI is the primary determinant of increased MEP during combined AO and MI^52^. Another hypothesis for the lack of AO + MI effect could also be the possible competition between the movement that the subjects imagined and the one they observed. As we asked the participants to perform kinesthetic imagery (i.e. to feel the movement) rather than visual imagery (i.e. to see the movement), the addition of action observation may have been counterproductive as the hand the participants had to observe was not their own. Moreover, there may be a visual dominance over kinesthesia^53^, so that the kinesthetic imagery may have been limited by the video.

### Facilitation of corticospinal excitability during mirror therapy performed alone or in combination with motor imagery

While the effects of MI and AO on MEP amplitude modulation have been widely investigated, this is not the case for MT. Few studies investigated MEP amplitude during MT in healthy participants and presented results that are consistent with those in the present study^2,28,33,54^. MT combines the observation of the movement in the mirror (visual feedback of movement) and a unilateral movement of the contralateral limb. While unilateral movement of the contralateral arm without any visual feedback has been reported to increase corticospinal excitability in the ipsilateral hemisphere^30^, likely due to interhemispheric connection between motor areas of the corresponding muscle via corpus callosum, a majority of studies did not find any increase in MEP amplitude during unilateral movement performed alone but only when visual feedback through a mirror was associated^29,33,55,56^. This supports the idea that interhemispheric connection may not play a crucial role in the effects of MT, at least during a simple task. Thus, increased corticospinal excitability during MT would be the consequence of the combination of visual feedback and unilateral movement, the commonly suggested hypotheses being a disinhibition of ipsilateral M1 provided by a reduced short-interval intracortical inhibition^29,56^ or activation of the mirror neuron system which in turn facilitates the excitability of M1^55^.

In the present study, we did not include a condition with contralateral movement. As such, we can only speculate on the respective influence of visual feedback and unilateral movement. Interestingly, the modulation of motor cortex excitability during ipsilateral movement only depends on task complexity, being stronger during complex movements involving several fingers compared to a simple repetitive tapping movement performed with a single finger^57^. It is also dependent on the level of contraction, being greater between 10–70% of maximum voluntary contraction than during a weak contraction^58^. Thus, considering the task performed by the participants in the present study, we hypothesize, but cannot rule out, that unilateral movement per se had no influence on corticospinal excitability. This could be further supported by findings from Tinazzi and al. (1998) demonstrating increased ipsilateral motor cortex excitability during complex fingers movements but not during a repetitive task consisting in opposition of the thumb and the third finger^59^ (i.e. the same movement as used in our study). We thus suggest that the effects of MT on corticospinal excitability were due to the combination of a visual feedback and a unilateral movement, at least in the context of the present study.

In our experiment, MT + MI also increased corticospinal excitability, as previously observed by others^25,28^. This may support the aforementioned hypothesis suggesting that the lack of corticospinal excitability modulation during AO + MI was due to a competition between kinesthetic imagination of participants’ own movement and observation of an external limb. Conversely, during MT + MI, the subjects had to imagine the movement in the same time they observed it as it was real, thus reinforcing their embodiment of the movement. Yet, the increase was not different than for MT performed alone, which is not in agreement with another report^28^. Once again, the discrepancy could be due to the lack of a real effect of MI in the present study. Thus, we suggest from our results that MT provided the strongest effects on corticospinal excitability in the context of our study, i.e. it consistently increased MEP amplitude when used in isolation or in combination with MI while MI did not potentiate the effects of AO or MT in opposition to our hypothesis.

## Limitations

A limitation of our study could be the fact that the number of MEP we analysed was different among conditions. For instance, it was significantly lower in the MT + MI condition than in control condition because more MEPs were discarded for this condition due to the presence of background EMG activity in some trials. However, such procedure was necessary to ensure no effects of EMG activity on MEP amplitude^37^. Another possible limitation to the present study is the fact that the intervals between trials were different between control and intervention sessions, i.e. seven sec between TMS pulses for the control condition vs one TMS pulse randomly delivered within each subset of three movement repetitions (one repetition being five sec in duration) for intervention conditions. While we cannot rule out that such difference may have influenced our results, this allowed us to avoid participants anticipation during intervention conditions^22^.

## Conclusion

In conclusion, we have compared corticospinal excitability modulation provided by MI, AO, MT, and the combination of MT + MI and AO + MI in healthy subjects. While no changes were observed during AO or AO + MI, we reported increased MEP amplitude during MI, MT and MT + MI when compared to a control condition. However, because MI provided no additional influence when combined with AO or MT, our results may suggest that, in healthy subjects, visual feedback and unilateral movement with a mirror may provide the greatest effects among all the tested motor simulations. Because MI, AO and MT are well known to provide benefits in neurorehabilitation, further studies are now needed to investigate the influence of combining different motor simulation strategies in this clinical context.

## Data Availability

All data are available from the corresponding author upon request.
